# From anatomical to biological resectability: navigating adaptive resistance in hepatocellular carcinoma conversion

**DOI:** 10.3389/fonc.2026.1849485

**Published:** 2026-07-22

**Authors:** Shitao Lei, Yun Cong, Yuhang Yang, Yaoqiang Bao, Wei Huang, Yingmei Shao

**Affiliations:** 1 Department of Hepatobiliary and Hydatid Disease Surgery, The First Affiliated Hospital of Xinjiang Medical University, Urumqi, China; 2 State Key Laboratory of Pathogenesis, Prevention and Treatment of High Incidence Diseases in Central Asia, Xinjiang Medical University, Urumqi, China

**Keywords:** clonal evolution, conversion therapy, hepatocellular carcinoma, minimal residual disease, tumor microenvironment

## Abstract

The advent of conversion therapy for hepatocellular carcinoma (HCC) marks a critical transition from palliative care to curative-intent surgery. While clinical success rates are rising, the molecular determinants of sustained remission remain inadequately defined. We propose that effective downstaging may indicate a comprehensive modulation of the tumor immune microenvironment potentially transitioning it from an immune-excluded to an inflamed phenotype based on early translational correlative data. However, the aggressive therapeutic selective pressure of targeted, immune, and locoregional therapies paradoxically fuels clonal evolution. In specific contexts, this therapeutic stress is hypothesized to drive treatment-resistant subclones through mechanisms such as Wnt/β-catenin signaling and metabolic adaptation. To navigate this therapeutic barrier, we advocate shifting the clinical objective from anatomical to biological resectability, operationally defined by a provisional set of measurable criteria including ctDNA clearance, robust CD8+ T-cell infiltration, and tertiary lymphoid structure maturation. By implementing a multidimensional surveillance framework that leverages liquid biopsies and AI-radiomics to track subclonal dynamics and minimal residual disease, clinicians can guide precise surgical interventions and intercept resistance, making a curative horizon attainable.

## 1 Introduction

Hepatocellular carcinoma (HCC) frequently presents with an indolent clinical course; as a result, a significant proportion of patients are diagnosed with advanced-stage disease. At this stage, high intrahepatic tumor burden or macrovascular invasion-particularly portal vein tumor thrombosis-renders curative R0 resection unfeasible under traditional surgical criteria (1–3). The synergistic integration of systemic agents, including tyrosine kinase inhibitors (TKIs) and immune checkpoint inhibitors (ICIs), with locoregional interventions such as transarterial chemoembolization (TACE) and hepatic arterial infusion chemotherapy (HAIC), has shifted the therapeutic objective. This multidisciplinary approach suggests a transition in the clinical focus from palliative containment toward a realistic pursuit of radical resection and long-term cure (4–6).

The clinical landscape for initially unresectable HCC has been fundamentally redefined by intensive combination regimens. By eliciting profound radiological or pathological responses, these conversion strategies have effectively extended the window for curative surgical intervention to a previously ineligible patient population. Emerging data suggest that survival outcomes within these responding cohorts are now beginning to converge with the prognostic benchmarks historically reserved for early-stage disease (7–16).

Despite these clinical gains, the orchestrating biological mechanisms remain incompletely defined. Evidence suggests that intensive conversion therapy imposes a divergent selective landscape on the tumor immune microenvironment (TIME). Primarily, it may galvanize an antitumoral state by promoting the neogenesis of tertiary lymphoid structures (TLS) and augmenting CD8^+^ T-cell infiltration-effectively activating previously immune-desert or suppressed tumors (17–19). Paradoxically, however, the acute treatment-induced selective pressure exerted by such regimens may facilitate clonal diversification. Current models hypothesize that by inducing metabolic adaptation and the compensatory recruitment of immunosuppressive populations, this therapeutic stress can select for highly plastic, treatment-resilient subclones. These subclones are implicated in orchestrating early post-surgical relapse in a subset of patients (17–24).

In light of this biological complexity, conventional anatomical downstaging constitutes an incomplete metric for the divergent clinical trajectories encountered in practice. Here, we synthesize the evolution of HCC conversion strategies, specifically dissecting the dynamic remodeling of the TIME and the molecular drivers of clonal resistance. We contend that the next frontier of therapy must transcend the current paradigm of anatomical resectability in favor of biological resectability-a framework that prioritizes the molecular signature of the tumor over its physical dimensions. By integrating the high-resolution insights of spatial multi-omics with the longitudinal monitoring capabilities of liquid biopsy, we propose a precision intervention framework to navigate the complexities of conversion therapy. Our objective is to delineate a rigorous, molecularly-informed roadmap toward curative-intent outcomes for patients with initially unresectable HCC, bridging the gap between current palliative standards and durable clinical recovery (22, 25). To explicitly delineate the robustness of current knowledge and clearly separate deterministic clinical outcomes from exploratory mechanistic inferences, this review categorizes cited evidence into four hierarchical levels: Phase III clinical trials (Level A evidence), Phase I/II trials (Level B evidence), translational/omics studies (Level C evidence), and hypothesis/modeling frameworks (Level D evidence).

## 2 Clinical paradigms: the strategic evolution of conversion therapy in HCC

From a clinical-translational perspective, conversion therapy represents a departure from traditional neoadjuvant protocols. While neoadjuvant therapy is typically applied to resectable cases to improve outcomes, conversion therapy is a strategic intervention designed to fundamentally alter the natural progression of the disease. By inducing profound downstaging, this approach renders patients-initially precluded from surgery due to excessive tumor burden or macrovascular invasion-eligible for curative-intent R0 resection or liver transplantation (7, 8). Consequently, the success of conversion is measured not just by tumor shrinkage, but by the restoration of a viable surgical window. During the initial era of systemic monotherapy, dominated by sorafenib and early-generation TKIs, objective response rates (ORR) remained suboptimal. Limited by a predominantly cytostatic profile rather than robust cytotoxic induction, these agents yielded conversion rates that largely stagnated in the single digits-exemplified by an ORR of approximately 8.4% for lenvatinib in pivotal trials (Level A evidence) -imposing a clinical ceiling on surgical salvage (26). This therapeutic plateau has been disrupted by the introduction of ICIs and their integration with locoregional modalities. These synergistic combinations have shifted the management paradigm toward a deep-response-oriented strategy, significantly expanding the possibilities for conversion in advanced HCC (3, 9).

### 2.1 Navigating high tumor burden: multimodal synergy and locoregional integration

While systemic combinations have recalibrated therapeutic expectations, achieving profound downstaging remains contingent upon overcoming the spatial and biological heterogeneity of high-burden disease. In refractory cases-particularly those characterized by bulky primary lesions, multifocal distribution, or portal vein tumor thrombus (PVTT)-systemic therapy alone may be insufficient to induce the requisite volumetric contraction within an optimal surgical window. Consequently, the integration of locoregional therapies has emerged as a critical adjunct, fostering a ‘triple-therapy’ paradigm. This approach leverages the immediate cytoreductive impact of local disruption to complement the sustained immunological and molecular control provided by systemic agents (12, 27). TACE is suggested to provide a dual-action mechanism that extends beyond simple mechanical devascularization. By inducing acute ischemic necrosis, TACE may serve as a potent catalyst for immunogenic cell death, precipitating a localized surge in tumor-associated antigens and damage-associated molecular patterns (DAMPs). This physiological insult essentially primes the tumor microenvironment, converting it from an immune-privileged site into an immunologically active milieu that is markedly more receptive to PD-1/PD-L1 blockade (12, 28). The clinical efficacy of this approach is underscored by data from the TACTICS-L trial and related investigations (Level B evidence), which demonstrate that the strategic sequencing of TACE with systemic TKI/ICI regimens significantly amplifies both ORR and survival durability in advanced HCC (29, 30). Complementing the embolization-based approach, HAIC exploits the hepatic first-pass effect to achieve sustained, supra-pharmacological concentrations of cytotoxic agents within the tumor and its associated feeder vessels. In the particularly recalcitrant setting of extensive portal vein tumor thrombus (PVTT, e.g., Vp4 type), the tripartite integration of HAIC, lenvatinib, and PD-1 inhibitors has demonstrated superior therapeutic synergy. This regimen has effectively extended survival compared to monotherapeutic standards, positioning it as the preferred conversion modality for this high-risk subset (13, 14, 31). Simultaneously, stereotactic body radiotherapy (SBRT) and Yttrium-90 (Y90) radioembolization have gained prominence as critical drivers of conversion. Evidence from the START-FIT and LEGACY trials-which reported conversion rates of 55% and complete response (CR) rates of 84%, respectively (Level B evidence)-underscores the capacity of radioimmunogenic modulation to reorganize the local microenvironment, thereby facilitating curative-intent outcomes (32–34).

### 2.2 The evaluation gap: reconciling radiographic remission with biological clearance

The clinical success of these potent conversion regimens has necessitated a recalibration of our evaluative frameworks. The transition from unresectable to resectable status is not merely a reflection of regressive anatomical dimensions; rather, it may indicate a profound attenuation of the tumor’s biological potency (35). Traditional RECIST 1.1 criteria, predicated on linear diametric measurements, frequently fail to account for the extensive intratumoral necrosis and treatment-induced edema characteristic of immunotherapy and locoregional disruption. This ‘radiographic-pathologic discordance’ can lead to a significant underestimation of therapeutic benefit. In contrast, modified RECIST (mRECIST) criteria—by prioritizing the assessment of the viable, contrast-enhancing tumor fraction—provide a more accurate proxy for true biological response compared to traditional linear measurements (36). However, it is imperative to critically recognize that mRECIST carries a significant potential to systematically overestimate downstaging in the setting of intensive combination therapy. The profound structural disruption caused by these regimens—such as treatment-induced inflammatory edema, extensive intratumoral necrosis, and the persistence of viable but non-enhancing malignant cells—can readily lead to false reassurance when relying solely on contrast enhancement patterns. Consequently, mRECIST alone is often insufficient to definitively discriminate viable tumor from complex treatment effects. To address this limitation, standard morphological assessment must be augmented by functional and molecular imaging modalities. Integrating techniques such as diffusion-weighted magnetic resonance imaging (DW-MRI), which detects hypercellular viable tumor regions through restricted water diffusion is crucial to better delineate true metabolic viability within the residual lesion. A critical clinical inflection point lies in whether radiographic remission serves as a faithful surrogate for pCR) which remains the most definitive correlate of RFS (37, 38). Complicating this evaluation landscape is the pronounced variability in pathological complete response assessment. The absence of a universally adopted pathological scoring consensus for quantifying treatment-induced necrosis versus microscopic viable residual tumor cells across different institutions introduces significant bias, confounding the comparative efficacy of various conversion regimens. Currently, a primary diagnostic bottleneck is the frequently observed imaging-pathology discordance: a subset of patients exhibiting radiographic CR may nonetheless harbor occult microscopic residual disease (MRD). This ambiguity drives a pivotal therapeutic tension between immediate surgical intervention-intended to ensure definitive oncological clearance-and a watch-and-wait strategy aimed at leveraging sustained systemic immunosurveillance (39, 40). The complexity of this decision-making is compounded by the divergent evolutionary outcomes of conversion therapy; while the regimen successfully debulks the dominant tumor mass, it simultaneously imposes a stringent selective pressure that may facilitate the survival of highly adaptive, treatment-resilient subclones.

### 2.3 Unresolved clinical challenges: heterogeneity, evidence gaps, and patient selection

Although the manuscript summarizes encouraging results of combination conversion therapies, a balanced critical appraisal must acknowledge the unresolved clinical issues that limit their universal application. Foremost is the lack of standardized criteria for patient selection for conversion therapy. Current clinical practices often conflate distinct baseline tumor burdens, macrovascular invasion statuses, and hepatic reserve parameters, leading to inconsistent conversion success rates and complicating the identification of ideal candidates (41, 42). Equally critical is the unresolved question regarding the optimal timing of surgery following conversion therapy. Currently, the optimal surgical window remains unstandardized. We emphasize that surgical timing should not be dictated solely by a fixed number of treatment cycles or a specific percentage of radiographic shrinkage. Instead, the decision to proceed to surgery must be dynamically determined through a comprehensive integration of maximal radiological response, adequate hepatic functional reserve, ctDNA/MRD status, AFP/PIVKA-II tumor marker kinetics, and the overarching risk of disease progression. Moreover, the landscape is characterized by marked heterogeneity among different combination regimens. The integration of systemic dual therapies (TKI plus ICI) with varying locoregional modalities (e.g., TACE, HAIC, SBRT) has created a myriad of protocols, yet there is a prominent lack of randomized evidence comparing these conversion strategies directly (43–45). Without Phase III randomized controlled trials to benchmark these heterogeneous approaches, therapeutic choices and sequencing remain empirically driven by institutional preference rather than high-level evidence, contributing to variable clinical trajectories.

## 3 Remodeling the TIME and orchestrating immune niches

**Figure 1:**
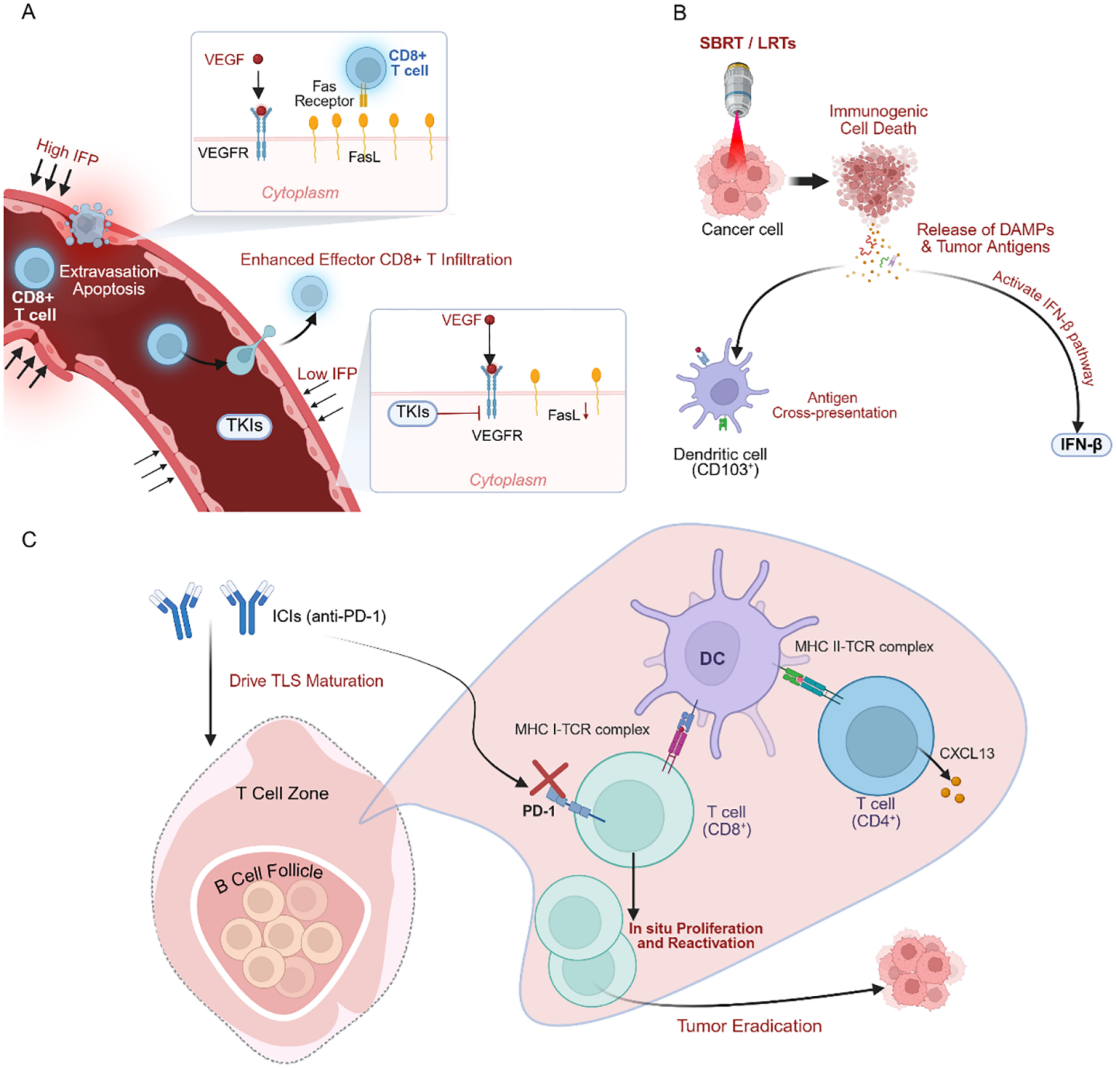
Conversion therapy-driven remodeling of the TIME in hepatocellular carcinoma. **(A)** Vascular normalization and T cell infiltration. Anti-angiogenic targeted drugs (TKIs) block VEGF/VEGFR signaling, thereby decreasing interstitial fluid pressure (IFP) and downregulating FasL expression on endothelial cells. This effectively reduces the apoptosis of CD8+ T cells during transendothelial migration, significantly enhancing the intratumoral infiltration of effector T cells. **(B)** Immunogenic cell death (ICD) and antigen presentation. Locoregional therapies (LRTs), such as SBRT, induce ICD in tumor cells, leading to the release of DAMPs and tumor antigens. This subsequently activates the IFN-β pathway and promotes antigen cross-presentation by CD103+ dendritic cells (DCs). **(C)** Tertiary lymphoid structure (TLS) maturation and microniche construction. Immune checkpoint inhibitors (ICIs) promote the maturation of TLSs, which encompass B cell follicles and T cell zones. Deep within the T cell zones, mature DCs, CXCL13-producing CD4+ T cells, and progenitor exhausted CD8+ T cells collaboratively form a functional cellular niche. Anti-PD-1 therapy alleviates the immunosuppression of CD8+ T cells and synergizes with microenvironmental signals to sustain their *in situ* proliferation and reactivation, ultimately achieving complete tumor clearance. Abbreviations: TKIs, tyrosine kinase inhibitors; ICIs, immune checkpoint inhibitors; LRTs, locoregional therapies; SBRT, stereotactic body radiotherapy; VEGF(R), vascular endothelial growth factor (receptor); IFP, interstitial fluid pressure; FasL, Fas ligand; ICD, immunogenic cell death; DAMPs, damage-associated molecular patterns; IFN-β, interferon-beta; DC, dendritic cell; TLS, tertiary lymphoid structure; MHC, major histocompatibility complex; TCR, T cell receptor.

**Figure 2:**
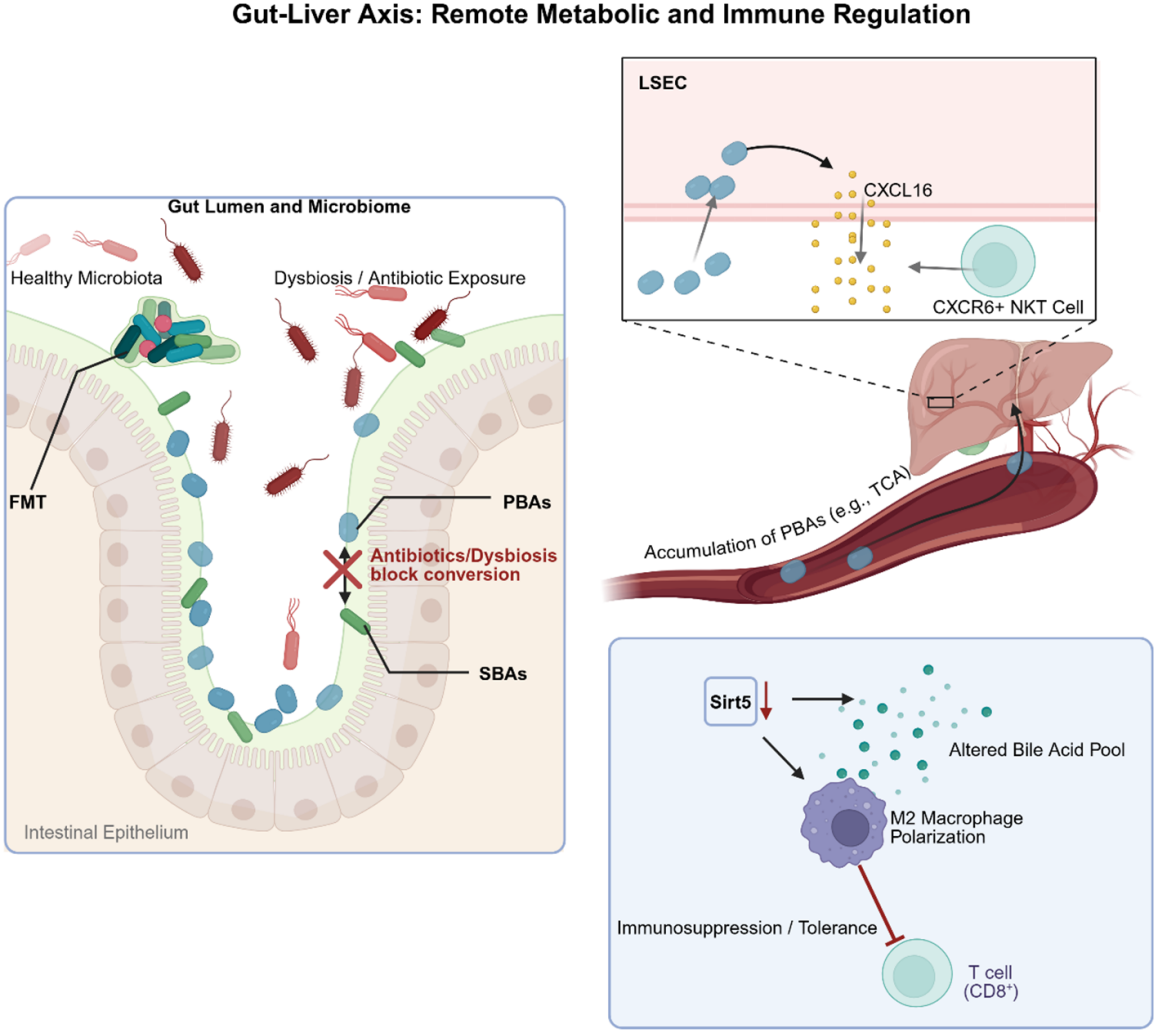
Distal metabolic and immune regulatory networks mediated by the gut-liver axis. The gut microbiota remotely regulates the immune status of the hepatic tumor microenvironment (TME) via bile acid metabolites. Under normal physiological conditions, the gut microbiota mediates the conversion of primary bile acids (PBAs) to secondary bile acids (SBAs). Anti-tumor sensitization axis (top right): When antibiotic exposure or dysbiosis blocks this conversion, it results in a substantial influx and accumulation of PBAs (such as taurocholic acid, TCA) in the liver via the portal vein. TCA stimulates liver sinusoidal endothelial cells (LSECs) to secrete the chemokine CXCL16, which subsequently recruits CXCR6^+^ NKT cells to infiltrate the tumor and induce apoptosis. Pro-tumor resistance axis (bottom right): Specific metabolic network imbalances (such as alterations in the bile acid pool triggered by Sirt5 downregulation) drive the polarization of macrophages toward the M2 phenotype. This subsequently suppresses CD8^+^ T cells, resulting in immune tolerance and resistance to conversion therapy. Clinical intervention strategies suggest that remodeling the gut microbial barrier via fecal microbiota transplantation (FMT) or short-chain fatty acid (SCFA) supplementation holds promise for reversing acquired resistance and resensitizing the anti-tumor immune response. Abbreviations: PBAs, primary bile acids; SBAs, secondary bile acids; TCA, taurocholic acid; FMT, fecal microbiota transplantation; LSEC, liver sinusoidal endothelial cell; NKT cell, natural killer T cell; Sirt5, sirtuin 5.

The success of conversion therapy hinges on reversing immune tolerance through a coordinated remodeling of the TIME. Systemic co-administration of anti-angiogenic TKIs and ICIs drives this transition. TKIs, such as lenvatinib or bevacizumab, prune aberrant vasculature and alleviate interstitial fluid pressure, which downregulates endothelial Fas ligand expression. This vascular normalization effectively prevents the apoptotic attrition of transmigrating CD8+ T cells, transforming a hostile milieu into a permissive landscape for immune infiltration (10, 22, 46–49). Beyond vascular stabilization, intensive regimens orchestrate the neogenesis of specialized immune niches essential for sustained antitumoral immunity. Spatial transcriptomic evidence (Level C evidence) highlights the maturation of tertiary lymphoid structures as pivotal immunological hubs that facilitate *in situ* B cell maturation and T cell priming (18, 19, 50). Furthermore, a localized cellular triplet niche—comprising mature regulatory dendritic cells, CXCL13+ CD4+ helper T cells, and progenitor exhausted CD8+ cells—serves as a critical nexus for sustaining T-cell proliferation and metabolic reinvigoration following PD-1 blockade (17, 51). Locoregional interventions (e.g., TACE, SBRT) further amplify this localized activation by inducing immunogenic cell death, releasing DAMPs, and priming CD103+ dendritic cells via the Type I interferon axis (12, 28, 32, 34, 52). The spatial and biological synergy of these therapeutic pillars is mechanistically detailed in **Figure 1**. Crucially, within the specific context of conversion therapy, this TIME remodeling is also subject to distal systemic regulation, primarily through the gut-liver immune axis. Gut microbiome-mediated conversion of primary to secondary bile acids acts as a systemic immune rheostat. The accumulation of primary bile acids (e.g., TCA) stimulates liver sinusoidal endothelial cells to secrete CXCL16, recruiting CXCR6+ NKT cells to the hepatic niche (53). To practically understand this distal regulation, **Figure 2** maps the gut-liver metabolic network. In the clinical timeline of conversion therapy, this distal regulation is inextricably linked to conversion success rates and surgical outcomes. For instance, the empirical use of broad-spectrum antibiotics during locoregional interventions (e.g., prophylactic administration post-TACE) can severely disrupt this delicate microbial balance, inadvertently dampening systemic ICI efficacy and lowering the probability of successful downstaging. Therefore, the clinical translational logic dictates that strictly auditing peri-conversion antibiotic exposure is vital. Furthermore, by proactively monitoring bile acid profiles or manipulating the microbiome (e.g., via fecal microbiota transplantation or short-chain fatty acid supplementation) during the pre-surgical conversion window, clinicians could potentially reverse acquired resistance, resensitize the anti-tumor immune response, and ultimately improve the rates of curative-intent salvage surgery. Thus, effective downstaging represents a multidimensional transition from an immune-excluded state to a highly organized, inflamed immune ecosystem.

## 4 Mechanisms of adaptive resistance and clonal escape

**Figure 3:**
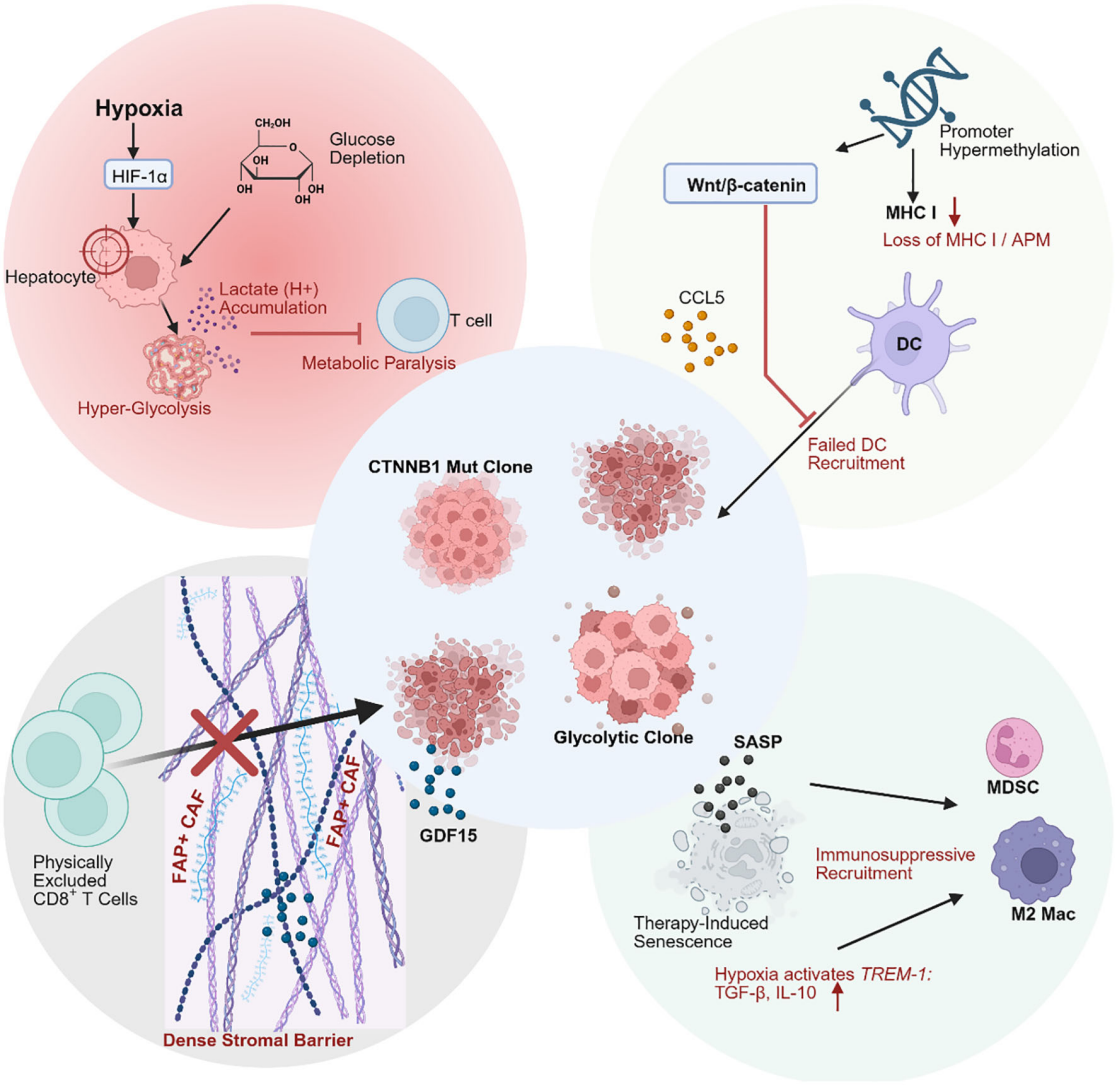
Adaptive resistance and multidimensional clonal evolution mechanisms under conversion therapy pressure. The treatment-induced selective pressure imposed by intensive conversion therapy eradicates sensitive clones while simultaneously driving the evolution of highly adaptive, drug-resistant subclones (e.g., CTNNB1-mutated or glycolytic phenotypes). Their escape mechanisms encompass four dimensions. Metabolic reprogramming (top left): The hypoxic microenvironment stabilizes HIF-1α to induce glycolysis, leading to glucose depletion and a massive accumulation of lactic acid (H^+^), which directly plunges CD8^+^ T cells into metabolic paralysis. Physical barrier and immune exclusion (bottom left): Fibroblast activation protein (FAP)-positive cancer-associated fibroblasts (CAFs) construct a dense collagen matrix that physically impedes T cell infiltration; meanwhile, the secretion of tumor-derived factor GDF15 induces an immune desert phenotype. Genetic and epigenetic escape (top right): CTNNB1 mutations drive Wnt/β-catenin pathway hyperactivation, blocking DC recruitment by downregulating the chemokine CCL5. Furthermore, promoter hypermethylation leads to the loss of MHC-I and the antigen processing machinery (APM), mediating immune stealth. Senescence and immunosuppressive networks (bottom right): Therapy-induced cellular senescence releases the senescence-associated secretory phenotype (SASP), which synergizes with the hypoxia-activated TREM-1 pathway (upregulating TGF-β and IL-10) to compensatorily recruit myeloid-derived suppressor cells (MDSCs) and M2 macrophages, thereby remodeling the immunosuppressive microenvironment. Abbreviations: HIF-1α, hypoxia-inducible factor-1 alpha; FAP, fibroblast activation protein; CAF, cancer-associated fibroblast; GDF15, growth differentiation factor 15; APM, antigen processing machinery; MHC-I, major histocompatibility complex class I; SASP, senescence-associated secretory phenotype; TREM-1, triggering receptor expressed on myeloid cells 1; TGF-β, transforming growth factor-beta; IL-10, interleukin-10; MDSC, myeloid-derived suppressor cell; M2 Mac, M2 macrophage.

Paradoxically, the intense selective pressure that drives TIME remodeling concurrently fuels clonal diversification, leading to acquired refractoriness. This adaptive resistance is governed by a sophisticated, convergent interplay of metabolic reprogramming, cell-intrinsic evasion, and structural barrier formation. Metabolically, treatment-induced hypoxia—particularly following aggressive embolization—stabilizes the HIF-1α axis, driving tumor cells toward accelerated aerobic glycolysis. The subsequent accumulation of lactate and glucose depletion bioenergetically paralyzes effector T cells (12). Simultaneously, this hypoxic stress activates the TREM-1 signaling axis and promotes the secretion of TGFβ and IL-10, orchestrating the secondary recruitment of highly suppressive MDSCs and M2-type macrophages to neutralize checkpoint blockade gains (54). Intrinsically, resistant subclones frequently hijack the Wnt/β-catenin pathway (via CTNNB1 mutations) to enforce immunological exclusion. Constitutive β-catenin signaling suppresses CCL5 transcription, crippling cDC1 dendritic cell recruitment and severing the link between innate sensing and adaptive immunity. Consequently, effector T cells remain physically sequestered at the stromal-tumor interface (55, 56). This spatial exclusion is reinforced by structural and epigenetic remodeling. Cancer-associated fibroblasts upregulate FAP to construct a dense, desmoplastic shield that physically precludes T-cell infiltration (48). Concurrently, tumor cells may undergo promoter hypermethylation of MHC Class I genes, resulting in epigenetic transcriptional silencing and a progressive loss of antigenic visibility (57). A critical, frequently overlooked dimension of this resistance is therapy-induced senescence. While residual malignant cells may exhibit a radiographic complete response by exiting the cell cycle, they remain metabolically hyperactive. These cells secrete a senescence-associated secretory phenotype, dominated by IL-6 and IL-8, which paradoxically fosters a supportive niche for the expansion of adjacent cancer stem cells and triggers an iatrogenic rebound (19, 58, 59). As comprehensively mapped in **Figure 3**, these four dimensions of acquired resistance—metabolic hypoxia, Wnt/β-catenin driven immune exclusion, dense stromal barrier formation, and therapy-induced senescence—frequently operate concurrently. This visual model provides a logical rationale for practical intervention, it dictates that post-conversion relapse is not a stochastic failure, but a structured evolutionary escape that fundamentally requires multi-target combination therapies rather than single-agent rechallenge.

## 5 Liquid biopsy and the molecular surveillance of treatment-selected resistance

The clinical challenge of the post-treatment evaluation phase is that these treatment-resilient subclones are often radiographically occult. Consequently, the integration of circulating tumor DNA (ctDNA) monitoring has emerged as the definitive tool for assessing MRD. Unlike traditional protein biomarkers like alpha-fetoprotein (AFP), which lack the sensitivity to detect low-frequency clonal expansion, ctDNA provides a high-resolution window into the genomic landscape of the surviving tumor. Quantitative longitudinal ctDNA analysis allows clinicians to detect molecular recurrence months before traditional imaging can visualize a lesion. Furthermore, by tracking the emergence of specific resistance mutations (e.g., CTNNB1 or TERT promoter mutations), liquid biopsy enables the real-time monitoring of the clonal sweep and provides the evidentiary basis for adjusting adjuvant strategies following surgical resection. Intratumoral heterogeneity functions as a latent reservoir for therapeutic resistance, with conversion therapy acting as the selective catalyst for large-scale clonal reorganization. Neoadjuvant intervention has been observed to initiate significant clonal shifts; early spatial multi-omic investigations of post-treatment specimens (e.g., following cabozantinib plus nivolumab) (Level C evidence) demonstrate that while therapy effectively eliminates dominant drug-sensitive lineages, it may simultaneously trigger the unrestricted proliferation of previously subordinate, resistant subclones. Freed from clonal competition, these resilient populations can undergo rapid expansion. In certain preclinical models (Level D evidence), these residual clones are typically characterized by distinct metabolic adaptations-specifically a shift toward oxidative phosphorylation-and the upregulation of progenitor-like stemness markers, such as CD44+ and EPCAM +. Emerging evidence supports that these therapy-selected populations may constitute the fundamental cellular seeds of postoperative recurrence (22, 60). Beyond clonal selection, the biological ontogeny of HCC recurrence follows distinct evolutionary trajectories. High-resolution single-cell sequencing has elucidated that true intrahepatic metastasis originates from the subclinical dissemination of primary tumor clones (Level C evidence), as opposed to multicentric (*de novo*) occurrence. This true recurrence is characterized by a definitive immune desert phenotype, which stands in stark contrast to the inflammatory milieu of the primary lesion. The orchestration of this immune-evasive state is primarily driven by the tumor-derived secretion of GDF15. Functioning as a potent mediator of immune tolerance, GDF15 subverts the adaptive response by directly impairing antigen presentation and facilitating the recruitment of lowly cytotoxic memory T-cell subsets. This paracrine signaling suggests the effective construction of a specialized niche that is refractory to immunosurveillance, providing a sanctuary for the expansion of metastatic clones (61).

## 6 Toward a biological definition of resectability: a precision oncology framework

The clinical trajectory of conversion therapy must be redefined: the goal is not merely the technical transition of a lesion from an unresectable to a resectable state, but the achievement of durable, long-term disease-free survival through systemic biological remodeling. To resolve the current discrepancy between anatomical success and oncological relapse, the field must undergo a paradigm shift from anatomical resectability to biological resectability. This framework moves beyond traditional volumetric assessment, anchoring surgical decision-making in the molecular fitness of the tumor and its microenvironment. To make this framework clinically actionable and testable in future prospective studies, we operationally define biological resectability as the achievement of a sustained, immune-permissive, and molecularly dormant state, which can be measured by a provisional set of integrated quantitative criteria. This state is primarily characterized by molecular clearance, evidenced by the sustained depletion or early kinetic clearance of ctDNA and the targeted eradication of high-risk clonal drivers, such as TERT or CTNNB1 mutations. Furthermore, it requires immunological conversion, defined by a quantitative threshold of robust CD8+ T cell infiltration into the malignant parenchyma, indicating the successful circumvention of stromal exclusion. Finally, this resectability must be anchored by architectural immune memory, wherein the definitive maturation status of tertiary lymphoid structures serves as an indispensable structural proxy for a self-amplifying and durable antitumoral memory. However, it is crucial to emphasize that universally accepted cutoff values for CD8+ T-cell infiltration and standardized grading systems for TLS maturation are currently lacking. Consequently, these immunological parameters must be rigorously evaluated as prespecified or exploratory endpoints in future prospective clinical trials, rather than being deployed as immediate diagnostic criteria for routine clinical decision-making. This transition is predicated on three strategic pillars: multimodal precision monitoring, biology-driven surgical timing, and the proactive management of clonal evolution throughout the perioperative continuum.

### 6.1 Beyond sensory limits: multimodal synthesis and defining clinical readiness

**Figure 4:**
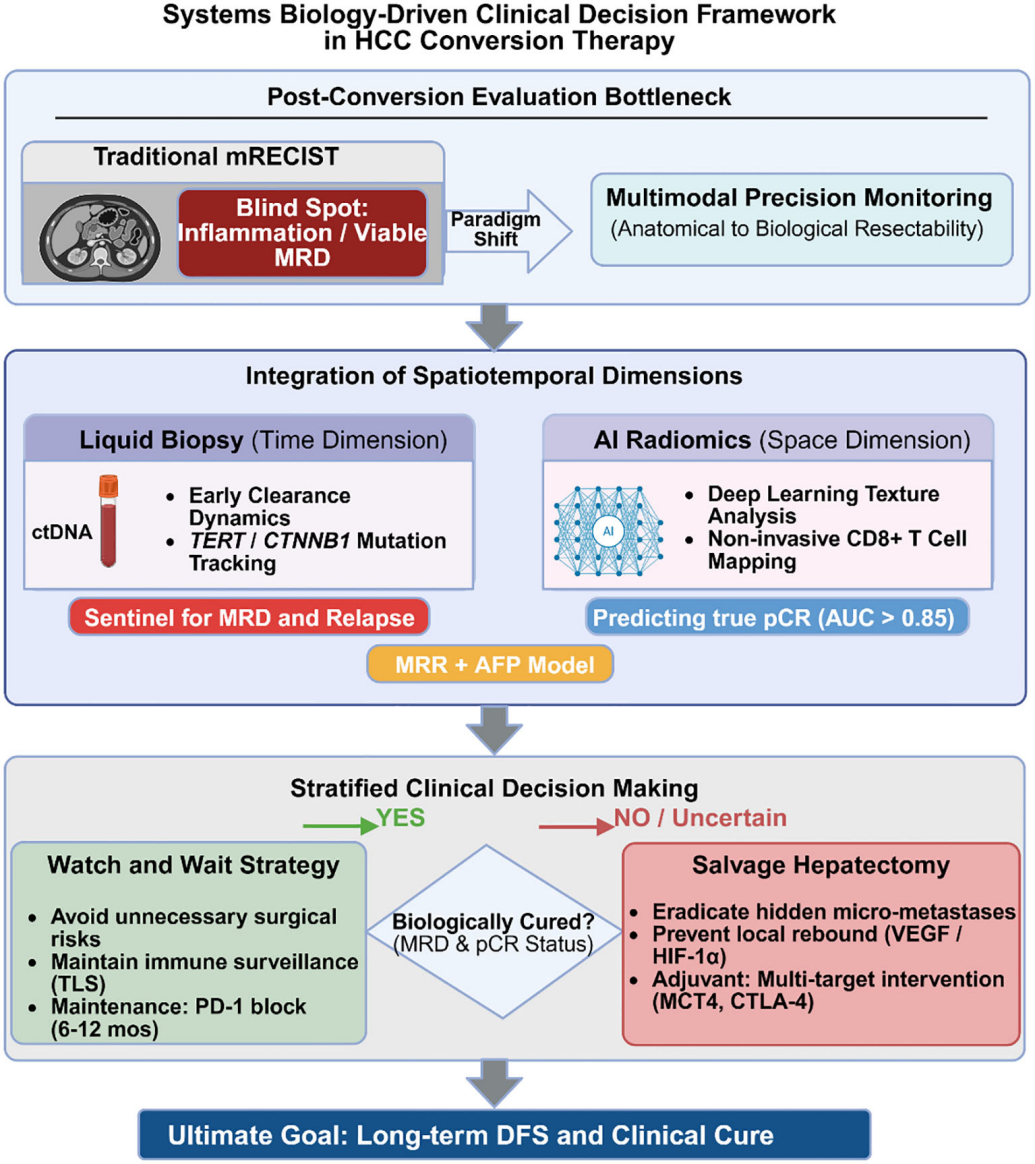
A clinical decision framework for conversion therapy in hepatocellular carcinoma driven by multimodal precision monitoring. This framework aims to achieve a paradigm shift from anatomical resectability to biological resectability. Post-conversion evaluation bottleneck: Traditional mRECIST imaging criteria possess evaluation blind spots regarding inflammatory edema and minimal residual disease (MRD). Spatiotemporal multidimensional integration engine: By integrating temporal liquid biopsies (tracking the early clearance of ctDNA and the dynamics of resistance mutations such as TERT/CTNNB1) with spatial AI radiomics (utilizing deep learning to analyze peritumoral textures and non-invasively map CD8^+^ T cell infiltration), combined with major radiologic response (MRR) and AFP models, true pathological complete response (pCR) can be predicted with high specificity. Stratified clinical decision tree: For patients achieving biological cure (MRD-negative and predicted pCR), an investigational watch-and-wait approach within strictly regulated trial settings may be considered to avoid unnecessary surgical risks and maintain PD-1 blockade to consolidate immune surveillance. Crucially, this investigational approach mandates stringent surveillance (e.g., high-resolution imaging and ctDNA tracking every 2–3 months) and predefined triggers for immediate crossover to salvage surgery (e.g., confirmed radiographic progression, dynamic rise in ctDNA/AFP kinetics, or *de novo* detection of resistance mutations). Conversely, for patients with biological residuum or indeterminate status, salvage hepatectomy is strongly recommended to eliminate occult micrometastases, supplemented by multi-target adjuvant interventions directed against resistance mechanisms (e.g., MCT4, CTLA-4). The ultimate goal of this proposed investigational framework is to provide an evidence-driven roadmap to safely navigate evolutionary bottlenecks and explore potential pathways toward a durable clinical cure. Abbreviations: HCC, hepatocellular carcinoma; mRECIST, modified Response Evaluation Criteria in Solid Tumors; MRD, minimal residual disease; ctDNA, circulating tumor DNA; AI, artificial intelligence; AUC, area under the curve; pCR, pathological complete response; MRR, major radiologic response; AFP, alpha-fetoprotein; TLS, tertiary lymphoid structure; DFS, disease-free survival.

Conventional imaging, governed by RECIST 1.1 criteria, frequently exhibits a response lag and fails to account for the phenomenon of pseudoprogression, while even mRECIST can be significantly confounded by treatment-induced necrosis and edema. Therefore, relying purely on anatomical or contrast-based metrics is insufficient for capturing residual tumor viability at the microscopic level. The path forward lies in a multimodal assessment framework that integrates functional imaging (e.g., DW-MRI, PET), liquid biopsy, and AI-driven radiomics to bridge temporal and spatial dimensions, resolving the inherent radiographic-biological dissociation. To operationalize this framework and address the translational gap, it is imperative to clearly delineate components that are currently ready for clinical practice from those remaining investigational. Currently, conventional protein biomarkers—primarily AFP, with PIVKA-II (also known as DCP) utilized where clinically available—remain the universally validated standards for baseline and post-treatment evaluation in clinical routines (62, 63). However, they lack the sensitivity to detect low-frequency clonal expansion or minimal residual disease (62, 63). To overcome this sensory limit, ctDNA monitoring, AI-driven radiomics, and spatial multi-omics are being actively evaluated. While these modalities predominantly reside in the investigational domain, they offer unprecedented resolution as genomic and spatial sentinels. However, the practical barriers to the widespread clinical adoption of liquid biopsy must be conspicuously acknowledged. Biologically, a substantial subset of HCC patients exhibits a low ctDNA shedding fraction, which inherently limits assay sensitivity. Technically and economically, the field faces a lack of standardized assays and uniform pre-analytical protocols, coupled with high costs and limited accessibility, especially in resource-constrained settings. To make this molecular surveillance framework more realistic and scalable, pragmatic solutions are essential. We propose the implementation of orthogonal validation strategies—such as combining ctDNA monitoring with conventional protein biomarkers (e.g., AFP or PIVKA-II)—to effectively compensate for low shedding fractions and minimize false negatives. Furthermore, rather than relying on broad genomic profiling, the development and deployment of cost-effective, HCC-specific targeted gene panels (focusing on high-yield resistance drivers) will be crucial to bridging the accessibility gap. For practical implementation, the longitudinal monitoring of ctDNA must be structured across defined temporal milestones rather than *ad-hoc* sampling. We advocate for a four-point assessment protocol. The first step is the baseline pre-conversion stage to map the initial clonal architecture and identify actionable targets. The second step is the early neoadjuvant phase to track the kinetic clearance dynamics of specific driver clones such as TERT or CTNNB1, which emerging evidence confirms significantly outperforms AFP in predicting pathological complete response, denoted as pCR, and long-term recurrence-free survival, denoted as RFS (62, 63). The third step is the preoperative window to definitively ascertain MRD status before surgical commitment. The fourth step is postoperative consolidation to longitudinally intercept early molecular relapse (62, 63). Simultaneously, investigational AI-driven radiomics serves as a non-invasive digital biopsy. By extracting high-dimensional textures, it visualizes intratumoral CD8+ T-cell infiltration density with an AUC greater than 0.85 to predict pathological responses to combined TKI and ICI regimens (Level D evidence) (64–66). Ultimately, by demonstrating the operational workflow for integrating longitudinal ctDNA tracking and AI-radiomics into clinical stratification, this multimodal approach transforms the surgical decision-making process from a reactive assessment of size into a proactive evaluation of biological fitness. As outlined in the decision tree in **Figure 4**, this framework provides an actionable algorithmic logic for clinicians: it explicitly guides the triage of patients achieving radiologic complete response into either a watch-and-wait cohort (strictly for MRD-negative and pCR-predicted cases) or a salvage hepatectomy cohort (for those with residual molecular persistence), thereby directly translating biological monitoring into precise surgical timing.

### 6.2 Resolving the decisional dilemma: interpreting pathoradiographic discordance

The establishment of precision predictive frameworks brings into sharp focus a critical decisional dilemma. For patients achieving a sustained rCR, clinicians must evaluate whether immediate salvage resection is superior to a disciplined watch-and-wait strategy. This conflict is rooted in the high frequency of pathoradiographic discordance, where mRECIST criteria frequently overestimate true therapeutic efficacy due to treatment-induced inflammatory edema or occult MRD masking viable malignant foci (39, 40). Resolving this clinical impasse necessitates a structured, scenario-based algorithm for interpreting conflicting radiological and molecular findings. Scenario 1 addresses Radiographic Remission with Molecular Persistence. When imaging suggests an rCR but genomic monitoring (e.g., ctDNA) remains detectable (MRD+), it signifies the occult persistence of therapy-resilient micrometastases (39, 40). Under this biological uncertainty, deferring surgery is hazardous; salvage hepatectomy remains the definitive gold standard to eradicate potential immune sanctuaries and preempt a localized rebound (39, 40). Scenario 2 involves Radiographic Residual with Molecular Clearance. Conversely, if imaging displays a residual mass but ctDNA is fully cleared alongside AFP normalization, the lesion often represents sterile necrosis or therapy-induced senescence rather than active disease (38, 67). To mitigate the risks of surgical overtreatment in this scenario, clinicians should rely on integrative models combining major radiographic response (MRR; >80% reduction in viable tumor volume) with biomarker kinetics (38, 67). Such synergistic models have demonstrated exceptional specificity (AUC > 0.90) in identifying true pCR (Level D evidence) (38, 67). Ultimately, a biological cure subcohort eligible for a watch-and-wait strategy can only be defined by the confluence of MRD-negativity and MRR fulfillment. However, we must strictly emphasize that the “watch-and-wait” approach in HCC currently remains an investigational paradigm rather than a standard of care. The existing evidence supporting active surveillance is largely derived from small retrospective series and propensity-matched analyses, which are inherently limited by substantial selection bias (38, 67). To avoid the profound risks of premature clinical adoption, this strategy should be stringently confined to highly regulated clinical trials. Within these investigational settings, we propose a rigorously defined minimum surveillance duration—involving high-resolution imaging and liquid biopsy tracking every 2 to 3 months for at least the first 24 months. Furthermore, explicitly predefined triggers for immediate crossover to salvage surgery must be established. These non-negotiable triggers include any confirmed radiographic progression, such as the expansion of the residual mass or the emergence of *de novo* intra/extrahepatic lesions, a consecutive dynamic rise in ctDNA or AFP kinetics, and the *de novo* detection of high-risk resistance mutations (e.g., TERT/CTNNB1). Crucially, the safety of deferring surgery should be further supported by investigational evidence of matured TLS, which provides the indispensable structural guarantee of a sustained, self-amplifying immune memory capable of maintaining long-term dormancy.

### 6.3 Mitigating clonal escape: post-resection consolidation strategies

Surgical intervention does not represent the terminus of the clinical journey, but rather a critical pivot point in the ongoing management of tumor clonal evolution. The high incidence of recurrence following even the most successful conversion surgeries underscores the necessity of a full-cycle longitudinal management strategy. To prevent the escape of occult, therapy-resilient clones, postoperative consolidation therapy is essential for maintaining systemic immunosurveillance. Current expert consensus recommends sustaining the effective conversion regimen (e.g., PD-1 blockade) for a minimum of 6–12 months post-resection. This strategy is designed to achieve two distinct biological objectives: first, the targeted eradication of MRD that may have survived the surgical intervention; and second, the preservation of the effector T-cell pool to prevent the exhaustion of the remodeled immune landscape (55, 68). Effective recurrence management necessitates a departure from static protocols toward dynamic therapeutic plasticity, informed by the specific mechanisms of emergent resistance. Post-conversion relapse signifies that the malignancy has overcome therapeutic selection, often acquiring novel escape traits such as Wnt/β-catenin pathway activation, metabolic reprogramming, or the upregulation of compensatory immune checkpoints. Under these conditions, the empiric re-administration of the index regimen is frequently futile. To navigate this clinical challenge, we propose a concrete, molecularly-guided salvage algorithm tailored to the specific resistance profile of the relapse clone. First, for oligometastatic or anatomically localized relapse, immediate locoregional salvage (e.g., SBRT or thermal ablation) should be prioritized to re-induce immunogenic cell death and act as an *in situ* vaccine, while maintaining or modifying the systemic backbone. Second, for immune-excluded relapse driven by Wnt/β-catenin activation or metabolic adaptation (identified via liquid biopsy or high-resolution spatial transcriptomics), continuing standard PD-1 blockade is insufficient. The optimal next-line strategy involves switching the TKI backbone to agents with broader multi-kinase activity—such as cabozantinib, which concurrently targets VEGFR, MET, and AXL to overcome acquired spatial exclusion—or deploying targeted metabolic inhibitors. Third, for relapses characterized by severe T-cell exhaustion, where the spatial microenvironment remains inflamed but functionally paralyzed, therapy must pivot to dual immune checkpoint blockade. The addition of a CTLA-4 inhibitor (e.g., ipilimumab) to the existing PD-1 backbone, or the integration of novel inhibitors targeting LAG-3 or TIM-3, is recommended to revitalize terminally exhausted effector populations (69, 70). Ultimately, conversion therapy is evolving beyond the pursuit of a surgical window; it is becoming a sophisticated undertaking in integrative precision oncology. By integrating longitudinal liquid biopsy surveillance, spatial multi-omics, AI-augmented radiogenomics, and multi-target precision interventions, we can transform the treatment of HCC from a reactive struggle against anatomy into a proactive, biologically-driven orchestration of long-term survival.

## 7 Conclusion

Conversion therapy-fueled by the mechanistic synergy of molecular, immunological, and locoregional interventions suggests a fundamental restructuring of the TIME, successfully bridging the clinical chasm between unresectability and radical surgery (9, 71). Nevertheless, the suspected emergence of treatment-resilient subclones-which studies suggest may be driven by Wnt/β-catenin activation, metabolic reprogramming, and therapeutic selective pressure-remains a primary barrier limiting long-term survival (22, 48). To elevate the objective of conversion from anatomical resectability to a definitive biological cure, the field must achieve three strategic paradigm shifts.

First, redefining screening criteria from anatomical feasibility toward biological benefit. We urge the field to evaluate conversion success through provisional measurable criteria—such as ctDNA clearance, validated CD8+ T cell infiltration thresholds, and TLS maturation status—to render biological resectability a clinically actionable endpoint. Future clinical decision-making must transcend mere radiographic assessment by integrating spatial multi-omics and liquid biopsy (ctDNA) monitoring. The core objective is the precise identification of biological responders, thereby circumventing futile interventions in cases characterized by systemic occult metastasis or high biological aggressiveness (22, 48). Second, innovating therapeutic strategies by constructing a multi-dimensional anti-resistance network. To counteract adaptive resistance, novel therapeutic dimensions must be explored: these include targeting aerobic glycolysis (e.g., MCT4 inhibition) to rectify metabolic immunosuppression (46), modulating the gut-liver axis microecology to potentiate immunotherapy (72); and introducing next-generation checkpoint inhibitors (e.g., LAG-3, TIM-3) to forestall immune escape following single-pathway blockade.Third, standardizing evaluative consensus to establish evidence-based benchmarks. The scientific community urgently requires a standardized definition of conversion success. This entails clarifying the prognostic weight of radiographic versus pathological responses and establishing optimized surgical window models that balance the risk of tumor progression against the benefits of matured immune memory (69).

The transition from unresectable HCC to a clinical cure is, in essence, an evolutionary competition between therapeutic intervention and malignant adaptation. Future conversion therapy must not terminate at the point of anatomical resolution; rather, it must be elevated into an integrative precision oncology approach. By decoding the molecular logic governing TIME remodeling and clonal evolution, we will finally translate the clinical promise of conversion into the durable reality of long-term survival.
